# Supplementary figures and images for: Microbiota-Dependent and -Independent Production of l-Dopa in the Gut of Daphnia magna
Source: mSystems. 2021 Nov 9;6(6):e00892-21. doi: 10.1128/mSystems.00892-21 (PMC8577283; doi:10.1128/mSystems.00892-21)

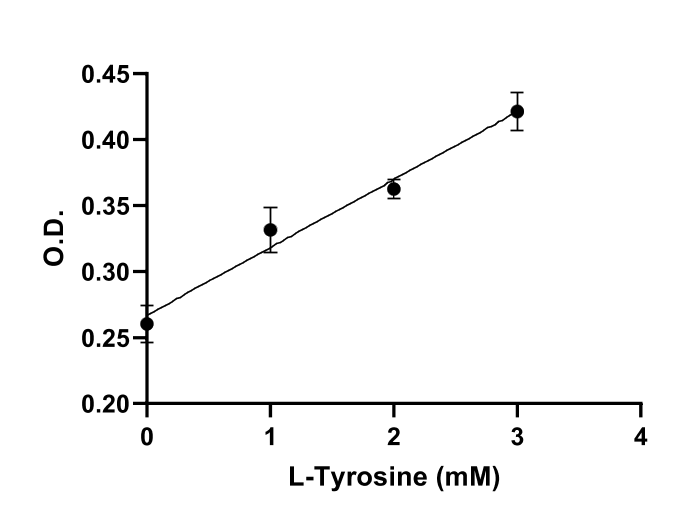

Supplement: FIG S1 [file msystems.00892-21-sf001.tif]

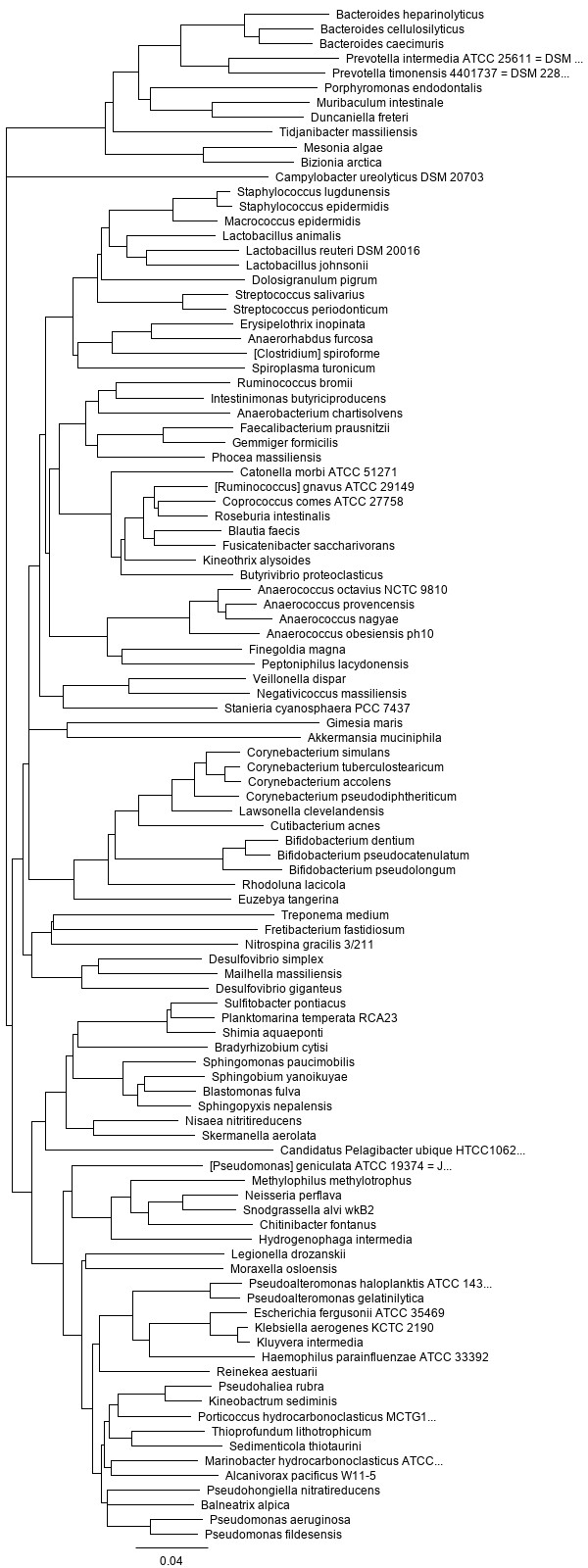

Supplement: FIG S3 [file msystems.00892-21-sf003.tif]

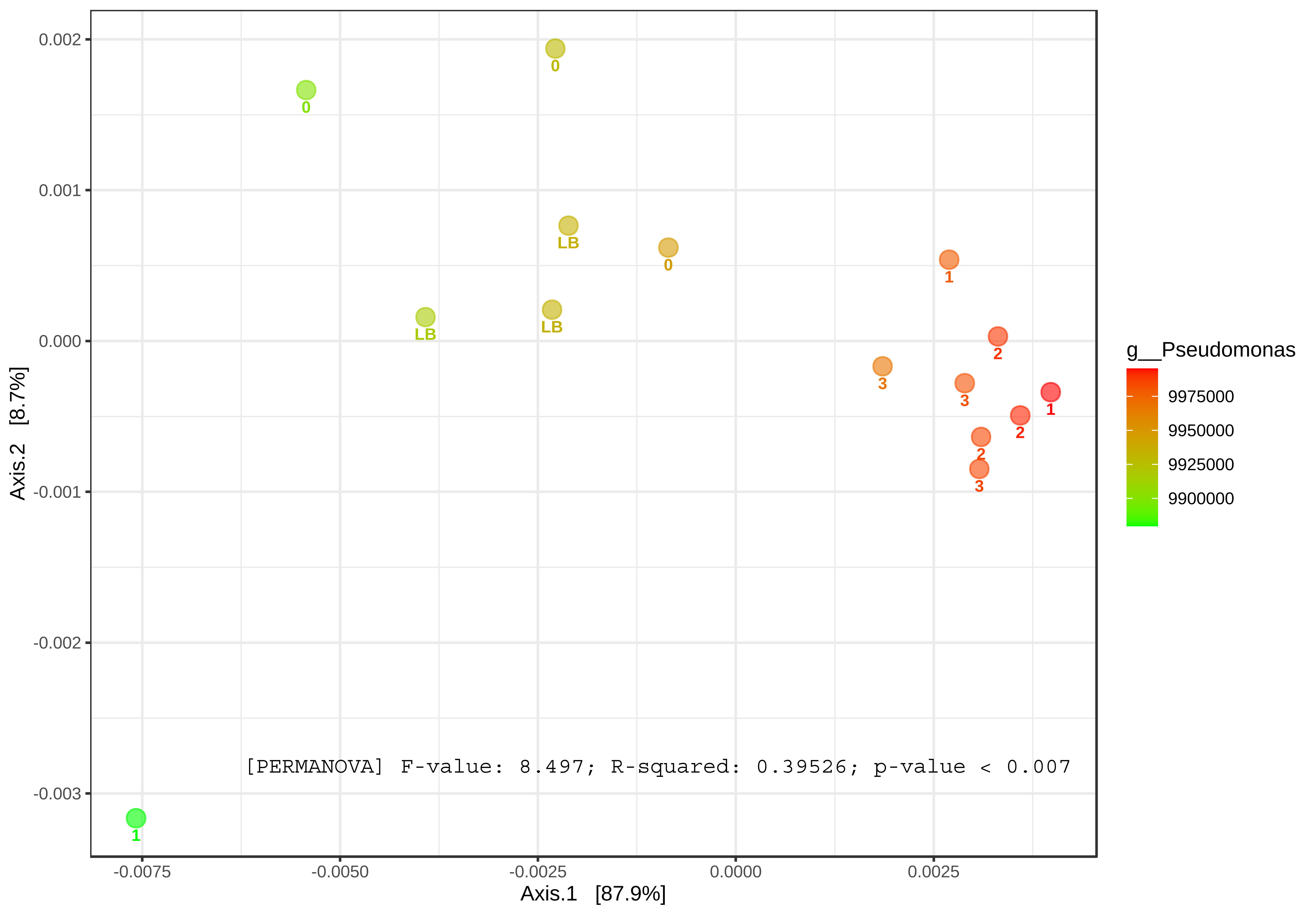

Supplement: FIG S4 [file msystems.00892-21-sf004.tif]

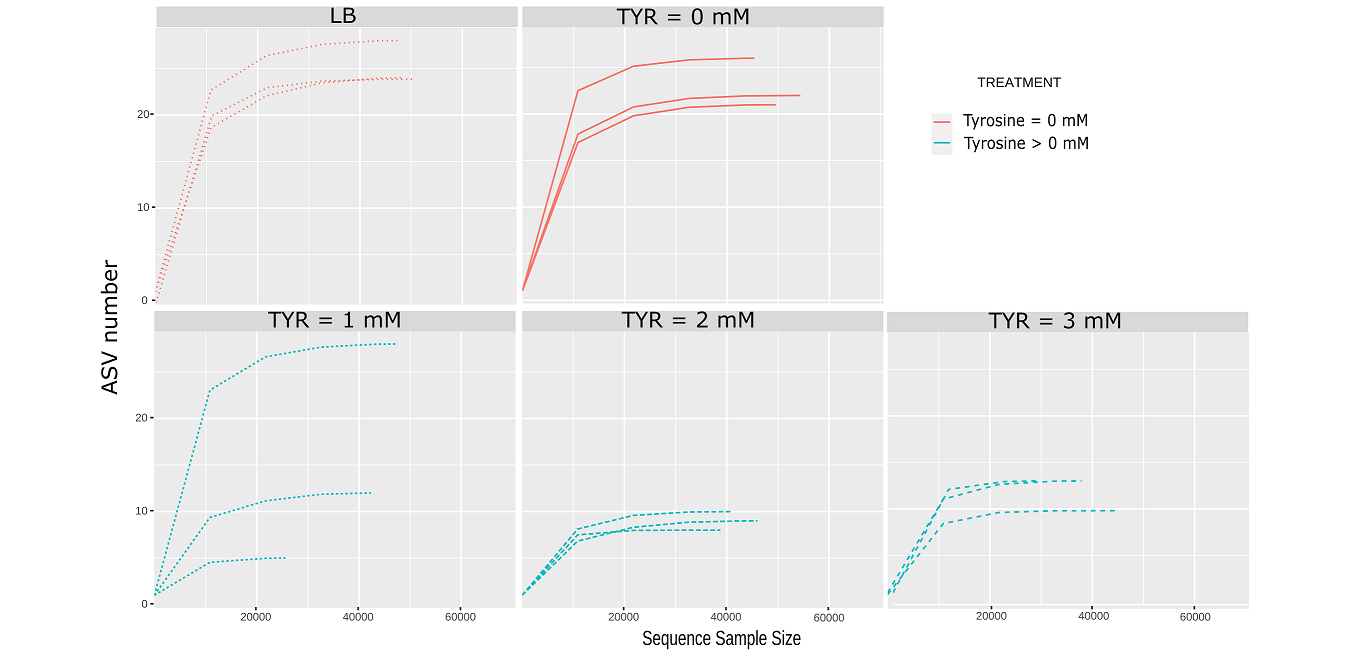

Supplement: FIG S2 [file msystems.00892-21-sf002.tif]
