# Supplementary material for: Microbiota-Dependent and -Independent Production of l-Dopa in the Gut of Daphnia magna
Source: mSystems. 2021 Nov 9;6(6):e00892-21. doi: 10.1128/mSystems.00892-21 (PMC8577283; doi:10.1128/mSystems.00892-21)
Supplement: TABLE S1 [file msystems.00892-21-st001.docx]

| Target gene | Forward primer sequence | Reverse primer sequence | Amplicon size (bp) |
| --- | --- | --- | --- |
| DDC | 5´-AAT GAT TCC TGA AGC CGC C-3´ | 5´-CCA GGC ATA ATG ACG CGT T-3´ | 81 |
| G3PDH | 5’-GAC CAT TAC GCT GCT GAA TAC G-3’ | 5’-CCT TTG CTG ACG CCG ATA GG-3’ | 100 |

*Campos, B., Rivetti, C., Tauler, R., Piña, B., and Barata, C. (2019). Tryptophan hydroxylase (TRH) loss of function mutations in *Daphnia* deregulated growth, energetic, serotoninergic and arachidonic acid metabolic signalling pathways. Sci Rep 9. doi:10.1038/s41598-019-39987-5.
